# Infant Hygiene in the Medical Curriculum

**Published:** 1914-05-02

**Authors:** J. Halford

**Affiliations:** Secretary. 4 Tavistock Square, London, W.C.


*To the Editor of* The Hospital.

### Infant Hygiene in the Medical Curriculum.

*To the Editor of* The Hospital.

Sir,—We should be greatly obliged if you would call attention in your Journal to the importance, from a public health point of view, of raising the standard of knowledge with regard to infant hygiene.

The National Association for the Prevention of Infant Mortality and for the Welfare of Infancy recently investigated the facilities which are at present afforded to medical students for instruction in infant hygiene. The investigation shows that in general the preventive aspects of infant hygiene are inadequately appreciated, and that sufficient attention is not given to the teaching of the home management of infants, and that the whole subject of infant hygiene holds too subordinate a position in the training of medical men as well as of nurses and midwives. The Association urges, therefore, that a course of clinical instruction on infant hygiene should be arranged by all the teaching centres, and that the certificate required from candidates in midwifery for the final examination should include evidence that they have received instruction in ante-natal pathology and hygiene as well as in the subsequent management of infants.—Yours faithfully, J. Halford, Secretary.

4 Tavistock Square, London, W.C., April 4.

